# Dental-dedicated magnetic resonance imaging in the follow-up of lower third molar removal

**DOI:** 10.1007/s11282-024-00787-x

**Published:** 2024-11-26

**Authors:** João M. C. E. S. Fuglsig, Matheus Sampaio-Oliveira, Rubens Spin-Neto

**Affiliations:** 1 Section for Oral Radiology and Endodontics, Department of Dentistry and Oral Health, Aarhus University, Vennelyst Boulevard 9, 8000 Århus, Denmark; 2 Department of Oral Diagnosis, Division of Oral Radiology, Piracicaba Dental School, University of Campinas (UNICAMP), Piracicaba, SP 13414-903 Brazil

**Keywords:** Third molar, Magnetic resonance imaging, Dental-dedicated magnetic resonance imaging, Aftercare, Case reports

## Abstract

The objective is to present a dental-dedicated magnetic resonance imaging (ddMRI)-based follow-up of inferior third molar removal over 12 months. A 30-year-old female presented with recurrent pain and bleeding from her lower right third molar. With adding diagnostic information from a panoramic image, the tooth was referred for removal. The patient underwent ddMRI using a dental coil with a proton density (PD) weighed turbo spin echo (TSE) sequence and a PD-TSE-STIR with fat suppression to highlight possible inflammatory processes. The scans were performed pre-operatively, immediately post-operatively, and in a rigorous follow-up (weekly basis for the first 6 weeks, bi-weekly from 7 to 12 weeks, and once at 6 and 12 months post-operatively). Using ImageJ software, circular ROIs were selected in the extraction alveolus coronary, middle, and apical regions. Mean grey values (MGVs) and standard deviation (SD) were obtained. A trend of decreasing MGVs in the PD (TSE) pulse sequence was observed over time, irrespective of the root third. Considering the PD-STIR (TSE), no trend was observed. ddMRI is feasible in the follow-up assessment of inferior third molar removal. Further clinical trials with larger samples are needed to define the usability of follow-up with ddMRI, considering a potential added diagnostic value.

## Introduction

The healing process of the alveolar bone after tooth extraction is of upmost importance for future rehabilitation (e.g., dental implant or prosthodontic replacement) and in cases when further intervention might be required [1]. The healing process takes place in different stages. First, the closure of the wound by soft tissues occurs (i.e., mucosa, coagulate and granulation tissues), and then a longer period by osteoid, bone formation, and calcification. The results of this process are well documented in both animal and human studies [2, 3]. It is widely accepted that without additional procedures, such as socket preservation [4] or socket-shield technique [5], this healing period is to be expected to last up to 6 months with the risk of pronounced reduction in bony dimensions [6]. Imaging of such processes in dentistry is limited to modalities based on ionizing radiation (i.e., X-rays) which show a momentary glimpse of the cortical and cancellous bone present in the exposed area (i.e., FOV), at the cost of ionizing radiation and the risk of stochastic cancer development [7].

Based on the current available methods, clinicians cannot usually predict immature or soft bone, dehiscence, fibrous areas, and other challenges upon re-entry for placement of dental implants (i.e., not immediate implant placement) even after several months of healing [8], which in turn can lead to the necessity of bone augmentation procedures, or even abandonment of the planned procedure if the circumstances are deemed unfavorable [9]. This is not always possible to detect on radiographs taken prior to surgery [10].

The ability to individualize the regime by which decision making and the timing of interventions is limited by radiation dose hygiene concepts, such as the ALARA (i.e., As Low As Reasonably Achievable) principle [11], and what can be depicted on X-ray based imaging. Dentistry is moving in a more individualized patient specific direction such as the introduction of the ALADAIP (i.e., As Low As Diagnostically Achievable being Indication-oriented and Patient-specific) principle [12], however, when imaging is indicated and what can be imaged is still limited by the same aforementioned shortcomings as has been the case for decades.

Dental-dedicated magnetic resonance imaging (ddMRI) is a novel proposed modality for imaging of the dental tissues [13]. Based on the tissues’ magnetic properties, rather than the ability to absorb X-radiation, this modality can be a suitable alternative for planning, executing, and following-up. Also, it could be helpful in the decision making and evaluation of tissues in the healing phases of treatment with its wider scope in tissue differentiation. Without the radiation burden from conventional X-ray based modalities, the possibility to obtain images of different tissue types (both soft and hard tissues) introduces the possibility to potentially individualize the monitoring of healing extraction sites in humans and evaluate bone formation and maturity prior to surgical intervention and during the surgical procedure.

The aim of this case report is to present a ddMRI-based follow-up of inferior third molar removal over the course of 12 months.

## Case report

**Fig. 1:**
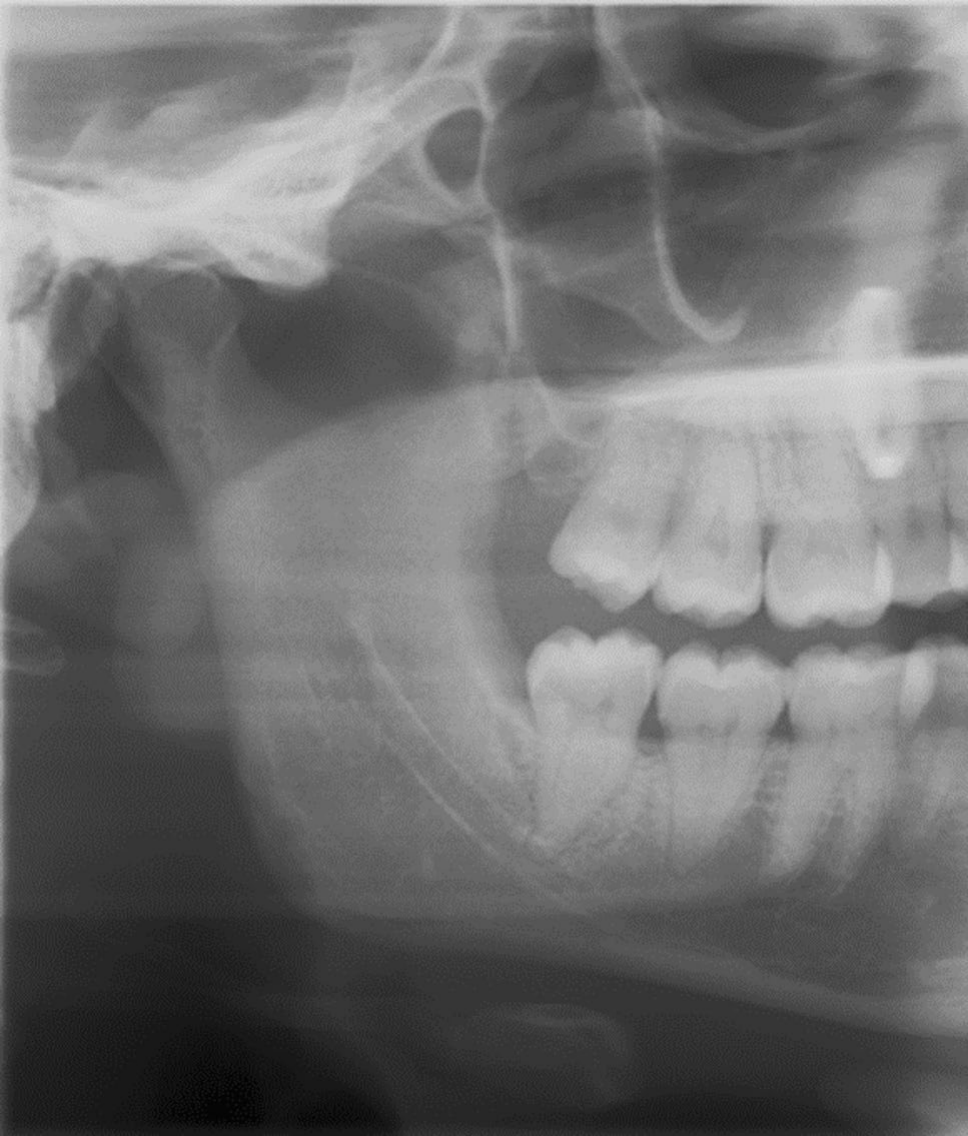
Right section of the panoramic radiograph taken 6 months prior to the extraction. The wisdom tooth antagonist visible in the radiograph was extracted shortly after the panoramic examination. A ghost artifact can be seen in the second premolar region of the maxilla due to failure to remove an earring prior to image acquisition

A 30-year-old female with no past medical, allergic or smoking history presented with recurrent pain and bleeding from her lower right third molar area. The patient had repeatedly undergone periodontal cleaning and had sublime oral hygiene, but experienced persisting pockets (> 5 mm) with bleeding on probing and periodically purulent fluid discharge. A recent panoramic radiograph was available (Fig. 1) and the lower right third molar was diagnosed to be vertically positioned and with a minor overlap of its fused apex in relation to the upper border of the mandibular canal, which could be indentified in its full extent. The tooth had no antagonist, as this had been removed 6 months prior due to a buccal position causing damage to the buccal mucosa at the time of the panoramic radiograph acquisition. The lower third molar was deemed to be worsening the periodontal prognosis for the adjacent second molar due to a horizontal bone loss. Adding the clinical and the radiographic information the removal of the lower third molar was indicated.

Prior to extraction the patient was informed of the possibility of scanning in a novel ddMRI machine (MAGNETOM Free.Max, Siemens Healthineers AG, Forchheim, Germany) which was recently installed at the Department of Dentistry and Oral Sciences (Institut for Odontologi og Oral Sundhed, Aarhus University, Aarhus, Denmark). The patient filled out an extended anamnesis check-up for MRI (Table 1) and was found to be a suitable candidate. Then, she was invited to undergo in post-operative follow-up. The patient was willing to participate based on an innate interest in the development of science and was offered no compensation for the time. The patient was not bound by any obligation to complete the proposed follow-up to comply with the specifications of the approval of the ethics committee evaluating the studies conducted at the site (Regional Ethics committee approval number: # 1-10-72-101-22).

**Table 1:** Extended anamnesis check-up used in the present case before ddMRI examination, showing the six categories and the respective questions

Category	Question
*Extended anamnesis check-up for ddMRI*	
1. Brain	Has the patient had metal inserted during neurosurgery?
	Has the patient had aneurysm clips inserted?
2. Ear	Has the patient had a metal artificial ear bone inserted?
	Does the patient have an intracochlear stimulator?
	Does the patient have a bone-anchored hearing aid?
3. Eyes	Has the patient ever had metal splinters in the eyes (e.g., from welding) that have not been removed?
4. Oral cavity	Does the patient have dentures or orthodontic apparatus?
	Does the patient have a denture with a magnetic attachment?
5. Heart and vessels	Has the patient had metal inserted during vascular surgery?
	Does the patient have a pacemaker or ICD device?
	Does the patient have any leftover pacemaker electrodes in the body?
	Does the patient have a mechanical heart or aortic valve prosthesis?
	Does the patient have heart monitoring (e.g., Medtronic Reveals)?
	Does the patient have implanted stents/aortic stents?
6. General	Does the patient have skin clips, clamps, Michelles clips, etc.?
	Does the patient wear an insulin pump, neurostimulator, or neuroshunt?
	Does the patient wear an artificial sphincter?
	Does the patient have a bladder catheter with a thermosensor?
	Does the patient have any other implanted metal parts/prostheses not mentioned above?
	Is the patient pregnant or suspected of being pregnant?
	Is the patient on hemodialysis?
	Is the patient claustrophobic?
	Does the patient have problems staying still for more than half an hour?
	Does the patient have piercings that cannot be removed?
	Does the patient have large tattoos?
	Does the patient have tattooed makeup?

The patient agreed to undergo ddMRI pre-operatively, immediately post-operatively, and a rigorous imaging regime for follow-up initially on a weekly basis for the first 6 weeks, then bi-weekly until 12 weeks, and finally once at 6 and 12 months post-operatively.

The extraction procedure was done under administration of 5.4 ml of local anesthesia (Xion® Inibsa Dental S.L.U. injection 20 mg + 0.0125 mg/ml epinephrine) as a mandibular nerve block and buccal infiltrations. Periodontal fibers were severed through the periodontal pocket and the tooth was luxated and elevated from the alveolus with a dental elevator seated in the proximal space between the third and second molars. The alveolus and distal portion of the second molar was cleaned for granulation tissue and calculus by means of manual curettage, and finally irrigated for debris with sterile saline water. No suturing was performed. Immediately post-operatively the patient was instructed to compress with sterile gauze for 20 min for initial hemostasis. The post-operative ddMRI scan was performed within an hour post-operatively. Post-operative pain management was administered according to Danish guidelines as 1 g of Paracetamol and 400 mg of Ibuprofen four times a day for 3–4 days depending on symptoms [14]. The patient reported no complications in the post-operative period.

### Image acquisition protocol

The scan procedures were performed by trained oral radiologists working with the ddMRI system. The patient was scanned in a supine position utilizing a 7-channel prototype dental surface coil (Dental Coil, RAPID Biomedical, Rimpar, Germany). A dedicated extraction sequence tree was followed for a total scan time of 8 min and 55 s. The examination comprised of a “scout”, low-resolution scan for identification of the ROI, followed by a series of multiplane 2D image acquisitions in the sagittal and coronal planes for imaging in higher resolution. These scans were performed with a proton density (PD) weighed turbo spin echo (TSE) sequence, and additionally a PD-TSE-STIR with fat suppression to highlight inflammatory processes in the alveolus and surrounding tissues. In Table 2, a detailed overview of the applied sequences is presented.

**Table 2:** Detailed parameters of dental-dedicated magnetic resonance imaging acquisition for each protocol

Pulse sequence	Planes	Reconstructed resolution (mm)	Acquisition time (min and s)	Basic scan parameters
PD (TSE)	2D (S, C, A)	0.3 × 0.3 × 2	2′38″	TR 2400 ms, TE 48 ms, FA 150°, bandwidth 100 Hz/pixel, GRAPPA acceleration (× 3), averages 4
PD (SPACE)	3D	0.4 × 0.4 × 0.7	3′09″	TR 750 ms, TE 29 ms, FA 170°, bandwidth 284 Hz/pixel, compressed sensing acceleration (× 4.2), averages 1.4
PD-STIR (TSE)	2D (S, C)	0.4 × 0.4 × 2.0	3′08″	TR 2560 ms, TE 46 ms, FA 130°, bandwidth 106 Hz/pixel, GRAPPA acceleration (× 3), averages 6

S = sagittal; C = coronal; A = axial

The planning of the sequences was done by hand attempting to mimic the previous angulation to the best of the operators’ ability utilizing the baseline post-operative scan as guide on a separate screen.

### Image export and assessment

**Fig. 2:**
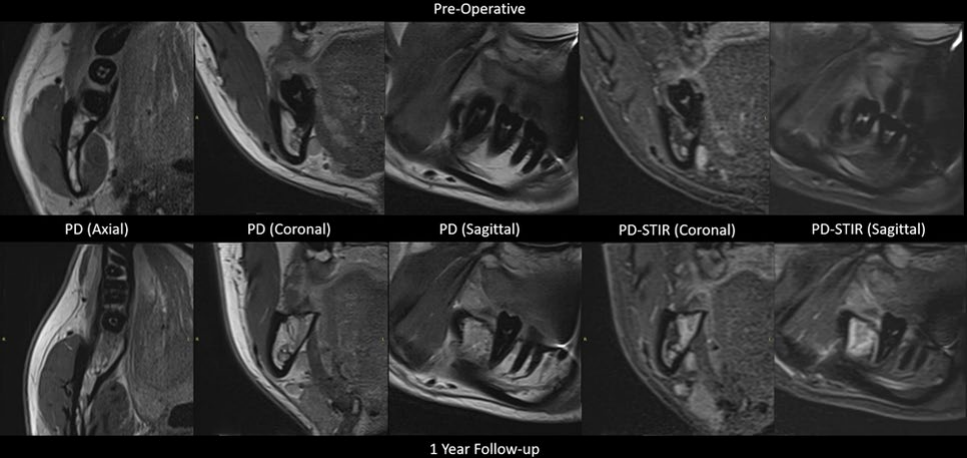
Corresponding image slices in various orientation planes pre-operatively (top row) and 1 year post-operatively (bottom row) in PD and PD-STIR

**Fig. 3:**
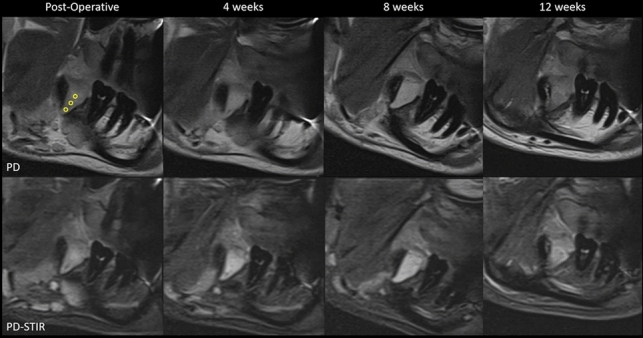
Corresponding image slices at four different times of the follow-up regime. On the top row displaying PD-weighted sagittal images, and on the bottom row PD-weighted images with STIR at weeks 0, 4, 8, and 12. An example of the three circular ROI (Ø = 4 mm, represented by yellow circles) that were used for mean grey value measurement across the follow-up regime

The patient was pseudomized according to the ethics committee protocol and DICOM volumes were exported to a safe university network drive. The image stacks were accessed in dedicated DICOM viewer software (RadiAnt DICOM viewer, Poznan, Poland) and corresponding slices were navigated using the coronal and sagittal orientation to find the corresponding 2D image/slice. The selected slices were then exported as portable network graphics files (PNG). Figure 2 shows selected image sections in PD and T2-STIR pre-operatively and 12 months after extraction, while Fig. 3 shows additional representative sagittal slices from the follow-up imaging regime from immediately after extraction, at 4, 8, and 12 weeks.

**Fig. 4:**
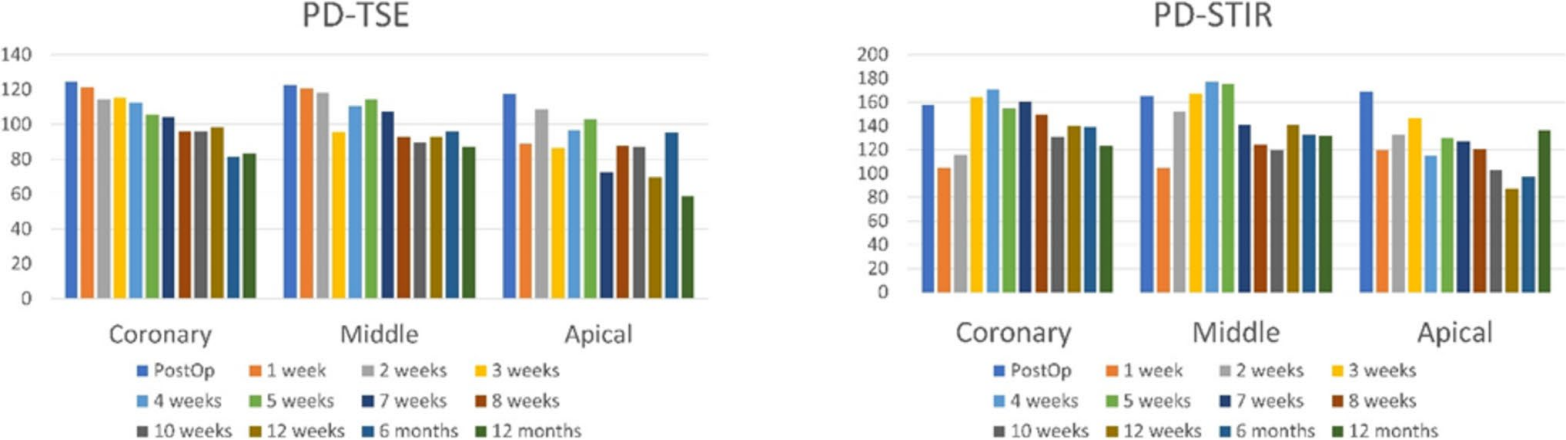
Bar graphs presenting the corresponding mean grey values according to the pulse sequence for each root third during the follow-up period

An objective analysis was performed using ImageJ software (ImageJ, National Institutes of Health, Maryland, USA). Circular ROIs of 4 mm in diameter were selected in the corresponding coronary, middle, and apical root thirds (Fig. 3). Mean grey values (MGVs) and standard deviation (SD) were obtained from each ROI for all images. Figure 4 shows bar graphs presenting the corresponding MGVs according to the pulse sequence for each root third and follow-up period. A trend of decreasing MGVs in the PD (TSE) pulse sequence was observed over the follow-up period, irrespective of the root third. Considering the PD-STIR (TSE with fat suppression), no possible trend was observed in MGVs over time. Regarding the SD, no trend was observed over time.

## Discussion

Inferior third molar removal is one of the most usual procedures in dentistry and requires imaging-based examination combined with clinical evaluation. According to the most recent guidelines regarding preoperative imaging for third molar removal, panoramic and intraoral radiographs are still the modalities of choice, whereas CBCT should only be used when the 2D imaging modalities do not fully cover the needs of the clinicians due to the higher costs and radiation for the patients [15].

Recently, ddMRI was introduced in dentistry and showed how MRI can be customized for dentomaxillofacial radiology by choosing the suitable magnetic field strength, specialized radiofrequency surface coil, and specific pulse sequences [13]. The system allows an MRI examination to be performed with a smaller carbon footprint, as resources such as electricity, water consumption, and Helium in the system are reduced, compared to the regular MRI units present in hospitals. Considering the elimination of ionizing radiation when working with ddMRI, this new imaging modality can reinvent the way a clinician might follow-up on cases of third molar removal, since the patient can be scanned as many times as necessary. Basically, a ddMRI scanning can be periodically performed, and in cases of any symptoms reported by the patients. This can lead to immediate intervention when needed.

Similarly to a CBCT scan, a ddMRI scan of lower third molars provides detailed information about the structure and configuration of the tooth and its roots, its positioning within the mandible, and its association with the mandibular canal. It also allows to verify the relationship with the mandibular second molar and detect bone loss between the second and third molars. Furthermore, some other relevant information which was not possible to obtain from CBCT scans is available in ddMRI scans, such as the visualization of the inferior alveolar nerve and lingual nerve [13].

Following tooth extraction, the wound healing process starts with the hemostasis. Then, an inflammatory phase takes place with neutrophils and macrophages clearing debris and bacteria. The next step is the proliferation phase, with fibroblasts producing collagen, forming granulation tissue, and new blood vessels. Finally, the maturation phase starts, with collagen remodulation, and scar tissue formation, with an increased strength and decreased vascularity. This process can last from months to years, depending on the extent of the wound [3]. Thus, considering the duration of the healing process, an extensive follow-up period was strictly adopted. Considering a ddMRI scan, the healing process is characterized by the darkening of the alveolus over time, suggesting the mineral accumulation within the tissues (i.e., calcification).

This case report showed how ddMRI could be used in the follow-up of third molar removal. Still, when looking at other applications related to the follow-up of the healing process, this imaging modality could also be used in other surgery-related procedures, such as the placement of dental implants. It is recommended to wait up to 6 months to place a dental implant after tooth extraction for sufficient bony healing [6]. However, this is based on a generalized mean time of healing. Using ddMRI, it would be possible to scan the patient multiple times, and the treatment planning could be adapted to each specific individual, leading to a more predictable treatment. When looking to possible limitations, a key challenge to consider in MRI is the presence of artifacts, influenced by both hardware and software. Higher magnetic field strengths increase the presence of artifacts, particularly near dental implants, due to magnetic susceptibility. This phenomenon causes local signal disturbances at tissue–material interfaces. Metal-containing dental materials create fewer artifacts at lower field strengths (e.g., the one used in ddMRI) compared to high field strength [13]. Further research is needed, regarding artifacts arising from common dental materials and to verify the feasibility of ddMRI assessments to determine the best and most relevant assessment periods and timing for placing a dental implant based on this new available information. It is also relevant to keep in mind that, the mere fact that ddMRI does not expose the patient to radiation does not suffice to justify that images should be acquired more often. The actual diagnostic impact of the images is yet to be determined, so no additional, unnecessary resources are spent. As MRI is a relatively new diagnostic modality to dentists, especially when used in a dedicated manner (i.e., ddMRI), future research in this novel field must create the basis on which to define guidelines of when and what to acquire images, always looking to benefit the patient.

An objective analysis was also performed in this case report to identify any possible relation of the post-operative period with changes in the MGVs on the extraction site. It was possible to observe a trend of decreasing MGVs in the PD (TSE) pulse sequence over the follow-up period, irrespective of the root third. This can be related to the previously mentioned “darkening” of the alveolus over time due to the healing process, which can be related to the calcification process. Another attention-grabbing finding was that no trends in MGV variation were seen for the PD-STIR (TSE with fat suppression). This is an interesting finding, suggesting no exacerbated inflammation-like reactions (e.g., edema) were present in the region over the assessed period. One can speculate that, in cases where post-operatory problems exist, this pulse sequence would be a good tool to screen for edema in the region. Another possible application of ddMRI during calcification processes could be the follow-up of healing process of endodontic lesions. Obtaining MGVs from ddMRI scans would be relatively easy for clinicians, so the method presented in this case report can be used in follow-up studies with a higher sample to be validated and applied in a clinical context.

In conclusion, this case report showed the application of ddMRI scans for follow-up of inferior third molar removal with trending MGV variation relatable to the expected calcification of the ROI. To the best of the authors’ knowledge, this is the first case report showing this usage. Further clinical trials with large samples are needed to define the usability of follow-up with ddMRI, considering a potential added diagnostic value. Also important to consider is the educational aspect, since clinicians and dental assistants need to be trained to work with this new imaging modality.

## Data Availability

Due to GDPR considerations and the nature of small sample case reports, the data for this study is not made publicly available.
